# Supplementary material for: Sex-Dependent Differential Expression of Lipidic Mediators Associated with Inflammation Resolution in Patients with Pulmonary Tuberculosis
Source: Biomolecules. 2022 Mar 24;12(4):490. doi: 10.3390/biom12040490 (PMC9025322; doi:10.3390/biom12040490)
Supplement: Supplementary file 1 [file biomolecules-12-00490-s001.zip › biomolecules-1623386-supplementary.pdf]

Supplementary material

Sex-dependent differential expression of lipidic mediators associated with inflammation resolution in patients with pulmonary tuberculosis

Claudia Carranza[2], Laura Elena Carreto-Binaghi[2], Silvia Guzmán-Beltrán[1], Marcela Muñoz-Torrico[3], Martha Torres[2], Yolanda Gonzalez[1], and Esmeralda Juárez[1*]

[1] Departamento de Investigación en Microbiología, Instituto Nacional de Enfermedades Respiratorias Ismael Cosío Villegas, México.

[2] Laboratorio de Inmunobiología de la Tuberculosis, Instituto Nacional de Enfermedades Respiratorias Ismael Cosío Villegas, México.

[3] Servicio Clínico de Tuberculosis, Instituto Nacional de Enfermedades Respiratorias Ismael Cosío Villegas, México.

* Correspondence to: Esmeralda Juárez, PhD. Calzada de Tlalpan 4502, Sección XVI, 14080 Tlalpan, Ciudad de México, México. ejuarez@iner.gob.mx

Supplementary Table S1. Demographic and clinical characteristics of the participants.

Characteristic	Patients, n=34	Healthy controls, n=27	p value*
Age, years, median (range)	45 (18-64)	32 (22-62)	**0.049**
BMI, median (range)	21.8 (12.7-39.7)	24 (20-30)	**0.005**
Leukocytes (10^3/mm3), median (range)	9.2 (4.1-15.2)	6.5 (3.6-10.6)	**<0.0001**
Neutrophils (10^3/mm3), median (range)	6.3 (2.2-13.5)	3.7 (1.2-7.6)	**<0.0001**
Lymphocytes (10^3/mm3), median (range)	1.3 (0.5-2.4)	2.2 (1.3-5.3)	**<0.0001**
Monocytes (10^3/mm3), median (range)	0.9 (0.1-1.5)	0.5 (0.3-0.7)	**<0.0001**
Eosinophils (10^3/mm3), median (range)	0.1 (0-10.1)	0.2 (0-0.5)	**0.036**
Basophils (10^3/mm3), median (range)	0 (0-0.2)	0 (0-0.1)	0.194
Erythrocytes (10^6/mm3), median (range)	4.4 (3.0 -5.5)	5.1 (4.2-6)	**<0.0001**
Hemoglobin (gr/dL), median (range)	12.5 (7.9-17.7)	15.7 (12.6-17.5)	**<0.0001**
Hematocrit (%), median (range)	37.7 (24-52.8)	45.8 (37.7-52.1)	**<0.0001**
Platelets (10^3/mm3), median (range)	341 (133-662)	220 (151-379)	**0.004**
Glucose (mg/dL), median (range)	133 (41-525)	95.3 (81-117)	**0.007**
Urea (mg/dL), median (range)	25.1 (10.7-57)	26.7 (12.8-42)	0.753
BUN (mg/dL), median (range)	11.7 (5-26.4)	12 (6-19.6)	0.923
Uric acid (mg/dL), median (range)	4.9 (1.5-13.7)	5.7 (3.3-10.4)	0.469
Creatinine (mg/dL), median (range)	0.7 (0.3-1.4)	0.9 (0.6-1.2)	**0.007**

*Fisher's exact test.

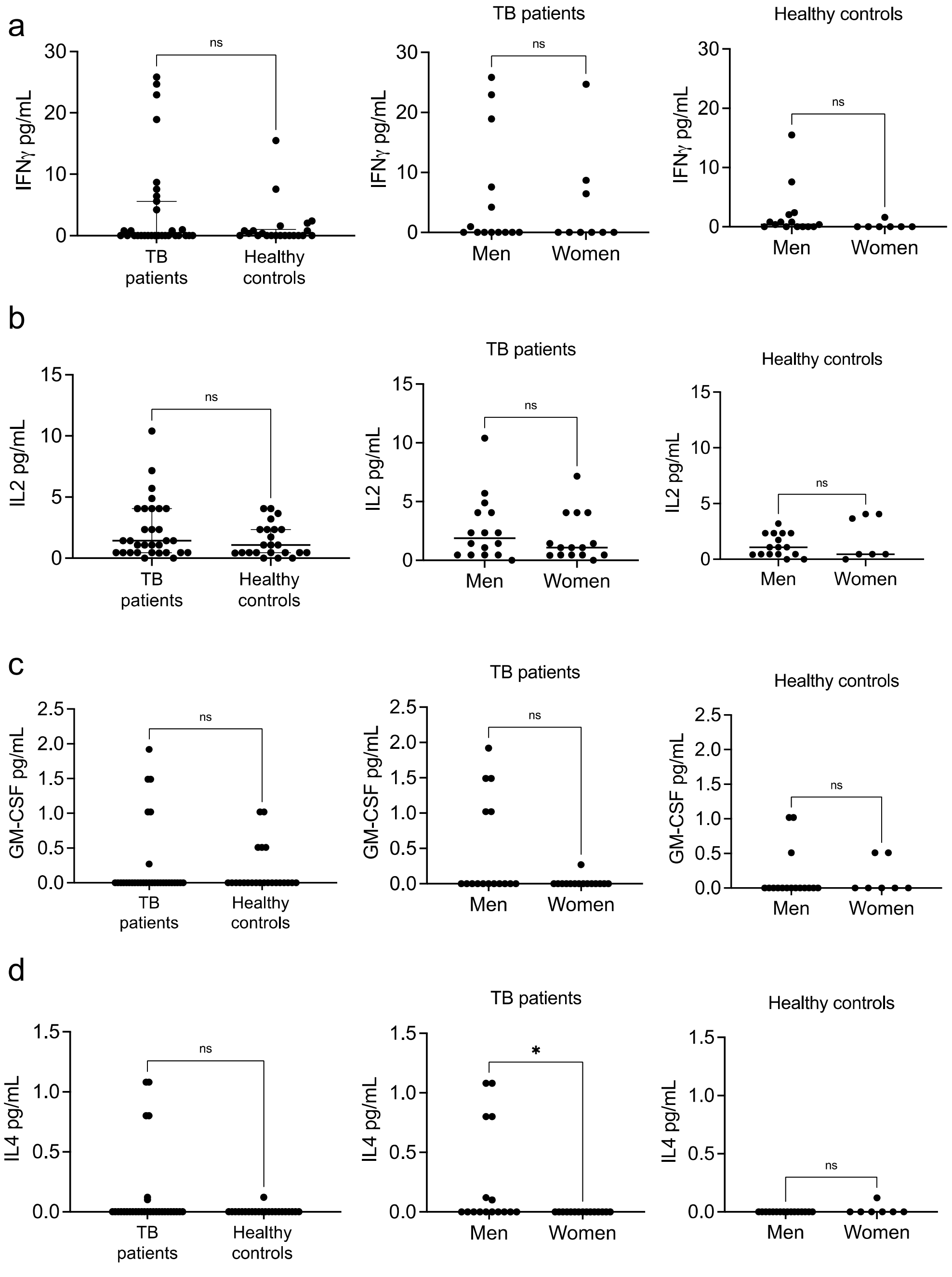

Figure S1. Additional circulating cytokines. Serum levels of IFNγ (a), IL2 (b), GM-CSF (c), and IL4 (d) were measured in patients with pulmonary tuberculosis and healthy controls using a Bioplex system. Individual results with medians and interquartile ranges are depicted (left). Data were disaggregated by sex for both the patients (center) and controls (right); depicted are individual results with medians. ns= not significant.

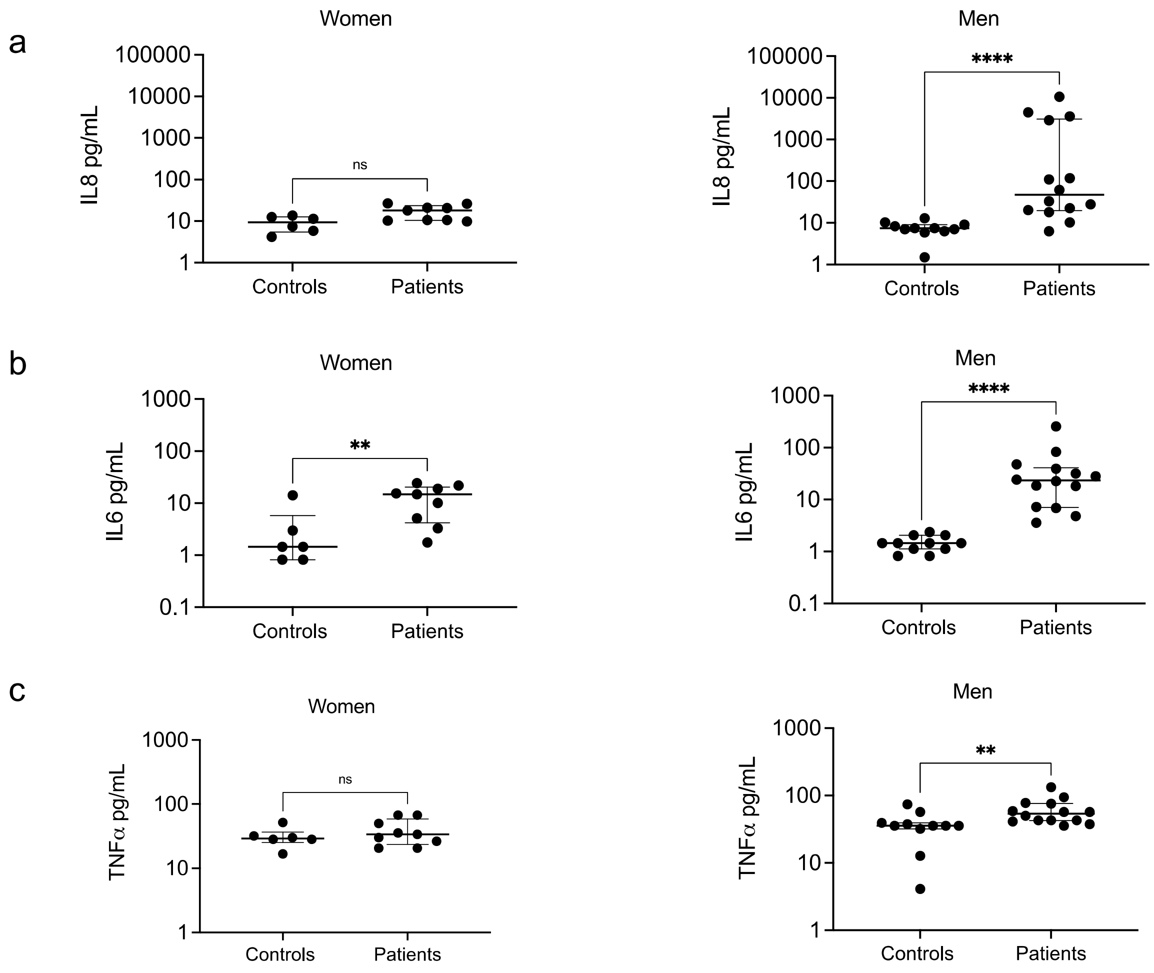

Figure S2. Male patients are the major contributors to the pro-inflammatory profile of serum cytokines in tuberculosis. Female patients have circulating levels of IL8 and TNFα similar to those of the healthy controls (left). All male patients had a clear pro-inflammatory cytokine profile (right). **p<0.01, ****p<0.0001, ns= not significant.
